# Antiretroviral therapy in people with HIV and end-stage kidney disease

**DOI:** 10.1097/QAD.0000000000004128

**Published:** 2025-02-04

**Authors:** Matthew Spencer, Christopher Pieri, Lisa Hamzah, Joyce Popoola, Sapna Shah, Rachael Jones, Jeremy Levy, Maurice Murphy, John Booth, Frank A. Post

**Affiliations:** a King's College Hospital NHS Foundation Trust; b Barts Health NHS Trust; c St George's Hospital NHS Foundation Trust; d King's College London; e Chelsea and Westminster Hospital NHS Foundation Trust; f Imperial College Healthcare NHS Trust, London, UK.

**Keywords:** antiretroviral therapy, end-stage kidney disease, HIV, kidney failure

## Abstract

**Objective:**

To summarize antiretroviral therapy (ART) use in the setting of end-stage kidney disease (ESKD).

**Design:**

Cross-sectional analysis.

**Methods:**

Descriptive analysis of ART regimens and dose of nucleoside/nucleotide reverse-transcriptase inhibitors (NRTI) in people with HIV and ESKD [dialysis, kidney transplantation, or estimated glomerular filtration rate (eGFR) <15 ml/min/1.73 m^2^] receiving HIV and renal care at five London centres. Exposures of interest were use of dual/unboosted ART regimens and higher than recommended doses of renally cleared NRTI.

**Results:**

A total of 157 participants were included (median age 55 years, 66% men, 84% black ethnicity, median CD4^+^ cell count 382 cells/μl, 99% HIV RNA <200 copies/ml). Fifty-eight (37%) were on dual/unboosted ART regimens, mainly dolutegravir/lamivudine. Participants on dual/unboosted ART had similar rates of HIV suppression as those on triple ART. Two participants currently virologically controlled on triple-ART had previously failed to suppress on dual/unboosted ART [dolutegravir/rilpivirine and dolutegravir/lamivudine (50 mg)]. Lamivudine doses were higher than recommended in 75 (77%) and lower than recommended in 8 (8%) participants. Full-dose lamivudine (300 mg daily) was used by 24 (32%) participants with eGFR less than 30 ml/min/1.73m^2^. None of those currently on reduced-dose lamivudine had required dose reductions for previous toxicity concerns.

**Conclusion:**

Dual/unboosted ART regimens, such as dolutegravir/lamivudine, provide robust viral efficacy in the setting of ESKD, and higher than recommended, including full-dose, lamivudine was well tolerated. The dolutegravir/lamivudine (300 mg) fixed-dose combination provides a single-tablet regimen for use across the eGFR spectrum, avoids under-exposure to lamivudine, and merits further evaluation in this population.

## Introduction

In 2023, an estimated 39 million people were living with HIV worldwide, mostly in Africa [1]. People of African ancestry worldwide are more likely to develop end-stage kidney disease (ESKD) from HIV-associated nephropathy (HIVAN) [2], with those of West-African ancestry including African Americans and Black Caribbeans disproportionally affected [3,4]. In most parts of Africa where access to kidney replacement services is nonexistent or highly selective, ESKD generally constitutes a terminal illness [5]. By contrast, in countries such as the United Kingdom, where access to renal replacement therapy and transplants are available free of charge, 1–2% of people of African ancestry with HIV are being dialysed or have received a kidney transplant, with favourable long-term outcomes [6]. Hence, substantial numbers of people with HIV and ESKD require antiretroviral therapy (ART).

Although ART continues to evolve for people with HIV with preserved kidney function, few studies of ART have been performed in individuals with ESKD, including recipients of kidney transplants. While European guidelines recommend avoiding drugs with nephrotoxic potential, such atazanavir, lopinavir, and tenofovir disoproxil fumarate (TDF), in individuals with impaired renal function, they do not provide recommendations for specific ART [7,8]. Integrase strand-transfer inhibitors (INSTI), which have high potency and few drug–drug interactions, have emerged as attractive agents for people with ESKD. Small, single-arm studies have generated some data in support of elvitegravir/cobicistat/tenofovir alafenamide (TAF)/emtricitabine (FTC), bictegravir/TAF/FTC, and dolutegravir/abacavir (ABC)/lamivudine (3TC) for individuals on haemodialysis [9–11]. Currently, there are no data to support the use of two-drug regimens, such as dolutegravir/3TC, in dialysis or kidney transplant populations.

Pharmacokinetic data suggest renally cleared nucleoside reverse-transcriptase inhibitors (NRTI), such as TDF, TAF, FTC, and 3TC, require dose reduction to avoid over-exposure in people with ESKD [12,13]. Clinical experience with full-dose FTC in people on dialysis, however, suggests increased FTC exposures are associated with little if any toxicity [9]. More recently, several studies have also suggested that 3TC can be used at full dose in individuals with estimated glomerular filtration rate (eGFR) greater than 30 ml/min/1.73 m^2^[14,15], and perhaps below this threshold [11]. In this study, we summarize ART experience including doses of renally cleared NRTI in a large cohort of people with HIV and ESKD.

## Methods

An ART utilization service evaluation was conducted in people with ESKD [eGFR <15 ml/min/1.73 m^2^ for >3 months or permanent renal replacement therapy (RRT; dialysis or kidney transplant)] who received HIV/renal care at five London centres between January and March 2024. We excluded individuals who were not engaged in HIV care or poorly adherent to RRT. As per local regulations, ethical approval and informed consent were not required.

We collected demographic and clinical information including kidney disease diagnosis, date of initiation and current mode of RRT. We recorded HIV parameters including current and nadir CD4^+^ cell count, HIV viral load, current ART regimen and dose of 3TC/FTC with start dates, hepatitis B/C co-infection status, presence of common comorbidities (hypertension, diabetes mellitus and cardiovascular disease: congestive cardiac failure, ischemic heart disease, stroke), current eGFR if not on dialysis. Use of transplant immunosuppressants, phosphate binders, proton pump inhibitors (PPI), or histamine type-2 receptor (H2) antagonists to evaluate for possible interactions with ART was also noted.

We stratified ART regimens into three a priori defined groups: triple/unboosted ART regimens containing three (unboosted) ART agents; dual/unboosted ART regimens containing dolutegravir with either an NRTI or nonnucleoside reverse transcriptase inhibitor (NNRTI); and boosted ART regimes containing a protease inhibitor or INSTI with ritonavir or cobicistat. For each participant on full-dose 3TC/FTC, we calculated follow-up on dual/unboosted ART regimens and full-dose 3TC/FTC following the onset of ESKD. We reviewed serial records to establish if those on a three-drug ART regimen had previously used and discontinued a dual/unboosted ART regimen, and whether those on reduced doses of 3TC/FTC had previously used full-dose 3TC/FTC in the setting of ESKD, and the reasons for abandoning these strategies.

## Results

After excluding nine individuals for poor engagement with HIV care and/or RRT, there were 157 participants with ESKD; their median age was 55 [interquartile range (IQR) 49–60] years; 66% were men, and 84% of black ethnicity (Table 1). Most had longstanding HIV and prolonged exposure to ART [16 (10–20) years], with a current CD4^+^ cell count of 382 (268–502) cells/μl and HIV-1 RNA less than 200 copies/ml. Ten participants (6%) had hepatitis B co-infection, and four (3%) past infection with hepatitis C. Most had longstanding kidney failure [median 6 (3–11) years], which was due to HIVAN in 62% (including focal and segmental glomerulosclerosis and arterionephrosclerosis). Eighty-three participants (53%) underwent dialysis (including 11 with a failed kidney transplant), 56 (36%) had functioning kidney allografts, and 18 had low eGFR but had not yet initiated RRT; the median eGFR in those with functioning allografts was 36 (27–50) ml/min/1.73 m^2^. Hypertension, diabetes mellitus, and cardiovascular disease were common comorbidities (present in 80, 31, and 22%, respectively), and PPI and/or H2-antagonists (54%) and phosphate binders (24%) commonly prescribed medications.

**Table 1:** Clinical characteristics of participants with end-stage kidney disease.

	All	Predialysis	Dialysis^a^	Transplant
	(*N *= 157)	(*N* = 18)	(*N* = 83)	(*N* = 56)
Demographic parameters				
Age	55 (49–60)	59 (50–63)	56 (48–60)	55 (50–59)
Gender (male)	104 (66)	16 (89)	59 (71)	29 (52)
Ethnicity				
Black (African/Caribbean/British)	132 (84)	16 (89)	65 (78)	51 (91)
White/other	26 (16)	2 (11)	19 (22)	5 (9)
Renal parameters				
Time since initiating RRT (years)	6 (3–11)	–	5 (2–10)	8 (4–12)
Current eGFR	31 (16–42)	11 (9–16)	–	36 (27–50)
Selected medications				
Transplant immunosuppression	67 (43)	–	11 (13)	56 (100)
Phosphate binders	37 (24)	1 (6)	34 (43)	2 (4)
PPI or H2 antagonists	85 (54)	6 (33)	39 (48)	40 (71)
Hypertension	125 (80)	17 (94)	62 (75)	46 (82)
Diabetes mellitus	49 (31)	7 (39)	24 (29)	18 (32)
Cardiovascular disease	35 (22)	2 (11)	22 (27)	11 (20)
HIV parameters				
Time since HIV diagnosis (years)	18 (12–22)	17 (13–21)	19 (12–22)	19 (12–25)
Time since starting ART (years)	16 (10–20)	16 (6–21)	17 (11–20)	17 (13–21)
Most recent CD4^+^ cell count	382 (268–502)	488 (230–566)	359 (268–470)	391 (281–518)
HIV RNA <200 copies/ml	156 (99)	18 (100)	82 (99)	56 (100)
Hepatitis B surface antigen (positive)	10 (6)	0 (0)	5 (6)	5 (9)
Hepatitis C antibody (positive)	4 (3)	0 (0)	2 (2)	2 (4)
Current ART regimen				
Dual/unboosted ART regimen^b^	58 (37)	8 (44)	33 (40)	17 (30)
Time on current ART regimens^c^ (years)	2 (1–4)	2 (1–3)	2 (1–4)	3 (1–4)
Unboosted (3/4 drug) ART regimens	81 (52)	8 (44)	39 (47)	34 (61)
Time on current ART regimen^c^ (years)	3 (1–6)	1 (1–3)	2 (1–5)	3 (1–6)
Boosted ART regimens^d^	18 (11)	2 (12)	11 (13)	5 (9)
Time on current ART regimen^c^ (years)	5 (3–8)	5 (3–6)	4 (2–8)	5 (5–7)

Data are expressed as median (IQR) or *N* (%). RRT, renal replacement therapy; eGFR, estimated glomerular filtration rate (CKD-EPI 2021); PPI, proton-pump inhibitor; H2, histamine type-2 receptor; ART, antiretroviral therapy.

a Mode of dialysis – haemodialysis: *N* = 80, peritoneal dialysis: *N* = 3; includes *N* = 11 with failed kidney transplants.

b Dolutegravir plus lamivudine, abacavir, or rilpivirine.

c Since ESKD onset.

d Regimens containing ritonavir or cobicistat.

### Antiretroviral therapy regimens in the setting of end-stage kidney disease

All participants were on ART; 58 (37%) were on dual/unboosted ART regimens, mainly dolutegravir/3TC (*N* = 50), the commonest regime overall, or dolutegravir/rilpivirine (*N* = 6). The median time on dual/unboosted ART regimens in the setting of ESKD was 2 (1–4) years, for a total of 134 person-years. A further 81 (52%) were on triple/unboosted ART, mainly ABC/3TC (*N* = 39) or TAF/FTC (*N* = 34) containing regimens, including 21 (14%) on TAF/FTC/bictegravir. The remaining 18 (11%) participants were on boosted ART regimens, mostly because of extensive resistance and/or intolerance to INSTI, and four were on intermittently administered TDF-containing regimens. The proportions of participants on dual/unboosted ART with HIV-1 RNA less than 200, less than 50 and less than 20 (100, 97, and 94%) were similar to those on triple/unboosted or boosted regimens (99, 94, and 90%). Four participants were no longer on dual/unboosted ART; two of these had developed virological failure with resistance, one on dolutegravir/rilpivirine (with concern regarding adherence and concurrent ranitidine use) and one on dolutegravir plus reduced-dose (50 mg) 3TC following a period of travel (Supplemental information).

### Dosing of lamivudine/emtricitabine and tenofovir alafenamide in the setting of end-stage kidney disease

There were 98 and 40 participants, respectively, on regimens containing 3TC and FTC. Table 2 shows the daily dose of renally cleared NRTI; only 15 participants (15%) received the recommended dose of 3TC for the degree of renal impairment; 75 (77%) received higher than recommended doses, with 300 mg daily (full-dose) used by 24 participants with eGFR less than 30 ml/min/1.73 m^2^, for a total of 56 person-years. No toxicity attributable to higher than recommended doses of 3TC was observed (Supplemental information). Of note, eight participants (8%) received lower than recommended doses of 3TC, all in the setting of a functioning kidney transplant. In those on FTC-containing regimens, all but one participant received full-dose FTC. TAF was used across the eGFR spectrum, including in six participants with eGFR less than 30 ml/min/1.73 m^2^ who were not receiving dialysis.

**Table 2:** Dosing of renally cleared nucleoside/nucleotide reverse transcriptase inhibitors in participants with end-stage kidney disease including kidney transplant recipients.

		Transplant or predialysis					Dialysis
	eGFR	60+	45–59	30–44	15–29	<15	–
Daily dose	3TC 25 mg	0	0	0	0	0	0
	3TC 50 mg	0	0	2	1	1	19
	3TC 100 mg	0	0	3	3	7	14
	3TC 150 mg	2	0	4	1	1	4
	3TC 300 mg	4	3	5	7	3	14
	FTC 200 mg	3	4	3	2	4	21
	FTC 200 mg BW	0	0	1	1	0	1
	TAF 25 mg (unboosted)	3	3	2	2	4	19
	TAF 10 mg (boosted)	0	0	1	0	0	2
	TAF 10 mg (unboosted)	0	1	0	0	0	0
	TDF 245 mg BW	0	0	1	1	0	1
	TDF 245 mg QW	0	0	0	0	0	1

Recommended dose of 3TC is highlighted in grey. 3TC, lamivudine; BW, twice weekly; eGFR, estimated glomerular filtration rate (CKD-EPI 2021); FTC, emtricitabine; QW, once weekly; TAF, tenofovir alafenamide; TDF, tenofovir disoproxil.

## Discussion

This study provides data on ART for a large cohort of people with HIV and ESKD for whom there is limited guidance regarding choice of ART. Lack of data and limited choice led to nonstandard ART regimens and considerable variation in practice. As 80% of participants had undergone or been recommended for kidney transplantation, ART regimens in this population should be optimized for the posttransplant setting, ideally avoiding boosted regimens that interact with transplant immunosuppression, rilpivirine (the absorption of which may be adversely affected by acid-suppression therapies), and TDF for its potential to cause renal toxicity.

Our data provide support for the use of INSTI-containing dual/unboosted ART (dolutegravir/3TC) and three-drug, unboosted ART regimens (TAF/FTC/bictegravir) in the setting of ESKD. As shown in people with HIV with preserved kidney function [16,17], viral suppression at viral load cut-offs of less than 200 copies/ml, less than 50 copies/ml, and less than 20 copies/ml with dual/unboosted ART regimens in our participants was similar to triple ART regimens. The two individuals who experienced virological failure raise, as in individuals with normal kidney function, concern about the robustness of dual/unboosted ART in individuals with suboptimal adherence and where dolutegravir/rilpivirine is coadministered with acid suppression therapy [18]. Additionally, marked 3TC dose reductions in the setting of kidney failure result in regimen complexity, which may affect adherence and suboptimal 3TC exposures when not regularly adjusted following successful kidney transplantation.

Our experience also suggests that higher than recommended doses of 3TC are generally well tolerated, provide opportunities for use of fixed-dose ART combinations and mitigate the risk of sub-therapeutic 3TC exposures posttransplantation. Our evaluation extends earlier studies, which were predominantly conducted in individuals with eGFR 30–50 ml/min/1.73 m^2^[11,14,15] to those with more severe CKD in whom standard 3TC doses will result in even greater exposures. Such exposures appear well tolerated; full-dose of 3TC was reduced in only one participant for possible toxicity when they developed pancytopaenia, but this was ultimately considered unrelated to the increased 3TC exposure.

The safety of TAF in those with eGFR less than 30 ml/min/1.73 m^2^, including kidney transplant recipients remains uncertain because of relatively high, free tenofovir exposures (5.7-fold increase in those with eGFR 15–29 vs. ≥90 ml/min/1.73 m^2^) that may impact residual kidney or graft function in those not requiring dialysis [19]. Hence, some clinicians continue to avoid TAF in this setting unless hepatitis B co-infection, prior HIV resistance or those in need of a fixed-dose combination to support adherence outweigh these concerns. This study did not set out to examine the relative safety of TAF in the setting of ESKD, but further studies may provide wider support for such regimens.

Finally, our study highlights how Black people with HIV, especially those of west-African ancestry, are disproportionally affected by kidney failure [3,4,20]. In these populations, genetic predisposition to kidney disease and HIVAN is mediated through variants of the *APOL1* gene [21], whereas sickle cell trait confers risk to milder forms of CKD [22].

Ours is the largest study on ART regimens in people with HIV and ESKD to date. We acknowledge several limitations, including the cross-sectional design, which may have reduced our ability to identify adverse outcomes with dual/unboosted ART regimens or high 3TC exposures; this was mitigated by retrospective review of ART regimens from the onset of stage 5 CKD to identify discontinuations of high-dose 3TC for clinical concerns. We did not include people who were poorly engaged in HIV or renal care or nonadherent to ART in whom dual/unboosted ART regimens are generally contraindicated, and our data may be affected by survivor bias. We did not collect data to examine for possible detrimental effects of TAF and TDF on kidney function in those not receiving dialysis. Finally, the average time on dual ART and full-dose 3TC was relatively short; hence, the possibility of toxicities associated with long-term over-exposure to 3TC cannot be ruled out.

In conclusion, dolutegravir/lamivudine and TAF/FTC/bictegravir provide robust viral efficacy to people with stage 5 CKD. Higher than recommended, including full-dose 3TC/FTC was well tolerated. The dual agent option of dolutegravir/lamivudine (300 mg) fixed-dose combination provides a single-tablet regimen for use across the eGFR spectrum, avoids under-exposure to lamivudine and over-exposure to tenofovir, and merits further evaluation in this population.

## Acknowledgements

The study findings have been submitted as an abstract for presentation at the British HIV Association Spring Conference (BHIVA, Brighton, 23–25 April 2025).

Author contributions: the study was designed by F.A.P., M.S., C.P., L.H., J.P., S.S., R.J., J.L., M.M., J.B. and F.A.P. provided clinical care to the participants and assisted with data collection. All authors interpreted the findings. M.S. and F.A.P. wrote the first draft of the manuscript. All authors revised and approved the final version of the manuscript.

### Conflicts of interest

J.L. reports personal fees from Gilead Sciences and ViiV Healthcare. M.M. reports conference sponsorship from ViiV Healthcare. S.S. reports consultancy fees from Astellas, Biotest, Takeda and GlaxoSmithKline. F.A.P. reports personal fees from Gilead Sciences, ViiV Healthcare/GlaxoSmithKline and MSD, and grants from Gilead Sciences, ViiV Healthcare/GlaxoSmithKline and MSD. All others declare no competing interests.

## Supplementary Material

Supplemental Digital Content
