# Supplementary material for: Antiretroviral therapy in people with HIV and end-stage kidney disease
Source: AIDS. 2025 Feb 4;39(7):863–8. doi: 10.1097/QAD.0000000000004128 (PMC12077330; doi:10.1097/QAD.0000000000004128)
Supplement: Supplemental Digital Content [file aids-39-863-s001.docx]

**Supplementary information**

**Additional demographic information**

Region of ancestry of Black participants (data available for N=96 (73%)

- West Africa 47 (49)
- East Africa 17 (18)
- Southern/Central Africa 20 (21)
- Caribbean 12 (13)

**Additional laboratory information**

**Nadir CD4 cell count**

- Available for N=89; median (IQR) 121 (31-226) cells/mm^3^

**HIV RNA**

- 148 participants (94%) had a VL <50 and 130/142 (92%) had VL <20 (at sites where VL was quantified to the <20 cut-off).
- 58/58 (100%), 56/58 (97%), and 48/51 (94%) on dual ART had VL <200, <50 and <20.
- 97/98 (99%), 92/98 (94%), and 82/91 (90%) on triple/boosted ART had VL <200, <50 and <20.

**HCV RNA**

- Four participants were anti-HCV positive; all were HCV RNA negative.

**Additional clinical information**

**Renal diagnosis**

- HIVAN/FSGS/HPT 98 (62%)
- Diabetic nephropathy 16
- IgA nephropathy 10
- Tubulointerstitial disease 8
- Immune-complex kidney disease 5
- Urological disease 4
- Adult polycystic kidney disease 4
- Thrombotic microangiopathy 2
- Unknown 10

**Renal replacement therapy status**

- Transplanted with functioning graft 56 (36%)
- Awaiting transplantation 27 (17%)
- Being worked-up for transplantation 42 (27%)
- Unsuitable (comorbidity/patient choice) 24 (15%)
- Not suitable for transplantation 8 (5%)

**Additional information on ART regimens**

**ART regimens**

- **Dual/unboosted ART regimens (N=58)**
  - Dolutegravir/3TC (N=50)
  - Dolutegravir/rilpivirine (N=6)
  - Dolutegravir/doravirine (N=1)
  - Dolutegravir/ABC (N=1)
- **Unboosted ART regimens (N=81)**
  - Bictegravir plus TAF/FTC (N=21)
  - Dolutegravir plus ABC/3TC (N=17) or TDF/FTC (N=2) or TAF/FTC (N=6)
  - Raltegravir plus ABC/3TC (N=9) or TDF/FTC (N=1)
  - Efavirenz plus ABC/3TC (N=7) or TAF/FTC (N=1)
  - Nevirapine plus ABC/3TC (N=5) or TDF/3TC (N=1)
  - Doravirine plus ABC/3TC (N=1) or TAF/FTC (N=2)
  - Rilpivirine plus TAF/FTC (N=1)
  - Other [Bictegravir plus TAF/FTC/Doravirine (N=2); Dolutegravir plus rilpivirine/3TC (N=1) or Doravirine/3TC (N=1) or Rilpivirine/atazanavir (N=1) or TAF/FTC/rilpivirine (N=1); Doravirine/3TC/raltegravir (N=1)]
- **Boosted ART regimens (N=18)**
  - Darunavir/cobicistat (N=1)
  - Darunavir/ritonavir plus 3TC (N=2)
  - Darunavir/ritonavir plus TAF/FTC (N=2) or Darunavir/cobicistat plus TAF/FTC (N=1)
  - Darunavir/ritonavir plus dolutegravir (N=1) or raltegravir (N=3)
  - Other [Darunavir/ritonavir plus dolutegravir/3TC (N=4) or raltegravir/ABC (N=1) or raltegravir/3TC (N=1) or maraviroc (N=1) or efavirenz (N=1)]

Abbreviations: ABC=abacavir, 3TC=lamivudine, TDF=tenofovir disoproxil, TAF=tenofovir alafenamide

**Individuals no longer on a dual/unboosted ART regimen (n=4)**

- **Case 1**: HIV was well controlled on weekly TDF plus DTG; ART was simplified to TAF/FTC/BTG to reduce tenofovir exposure while maintaining hepatitis B cover.
- **Case 2**: HIV was well controlled on ABC plus 3TC plus DTG; ART was simplified to DTG/3TC but patient was intolerant (suspected intolerance to an excipient in the fixed dose combination); her ART was switched to DTG/DOR/3TC while the viral load remained suppressed.
- **Case 3**: HIV was well controlled on AZT/3TC/EFV (intolerant to ABC; 3TC dose adjusted for renal function); ART was changed to DTG/RPV due to anaemia. She developed treatment-emergent resistance (K101E and Y181C) after five months of dual ART (viral load 980 copies/mL); ART was switched to TAF/FTC/DTG with re-suppression of HIV RNA. Of note, patient had low mood, a history of suboptimal engagement with HIV care and adherence to ART, and she may not always have adequately spaced RPV and ranitidine doses.
- **Case 4**: HIV was well controlled on TAF/FTC/DTG; ART was changed to DTG/3TC due to eGFR decline, with 3TC dose adjustment for renal function from 150 to 50 mg daily. He disengaged from care for more than one year and required emergency dialysis abroad. On return to the UK, he was pre-emptively switched to TAF/FTC/DTG and his viral load found to be 200 copies/mL with M184V on resistance testing. The total duration of DTG/3TC was 35 months; it was felt that underexposure to 3TC had contributed to dual ART failure.

**The total cumulative exposure to full dose lamivudine in our participants with stage 5 CKD was 93 person-years, including 56 person-years in those with current eGFR <30. At the time of this analysis, 2 individuals were no longer on full dose lamivudine:**

- Lamivudine dose was reduced in one participant with unexplained pancytopenia in the setting of general clinical deterioration resulting in death; dose reduction did not result in improvement of haematological indices; no cause for the pancytopenia was found; no post-mortem was performed.
- Lamivudine dose was reduced in one participant in whom the ART regimen was changed from a boosted to an unboosted regimen, with concomitant dose reduction of lamivudine.

**Reasons for use of tenofovir and boosted protease inhibitors**

- N=4 study participants were on tenofovir-DF. 2 participants were on TDF to provide hepatitis B cover, 2 participants were on TDF due to HIV resistance.
- N=37 participants were on regimens containing tenofovir alafenamide: 9 to provide hepatitis B cover, 9 due to resistance, 2 due to poor adherence, 1 due to no response to the HBV vaccine.
- N=18 participants were on boosted regimen: in 12 due to HIV resistance, 2 due to side effects form other ART, and a result of patient choice in 1; in 3 the reason was unclear.
